# The Keto Functions of Heme *d*_1_ Are Introduced by NirF and NirC

**DOI:** 10.1002/cbic.70370

**Published:** 2026-05-13

**Authors:** Pia Hebecker, Gunhild Layer

**Affiliations:** ^1^ Institut für Pharmazeutische Wissenschaften Pharmazeutische Biologie, Albert‐Ludwigs‐Universität Freiburg Freiburg Germany

**Keywords:** enzymes, heme *d*_1_ biosynthesis, high‐performance liquid chromatography, NirC, NirF, tetrapyrrole

## Abstract

The modified tetrapyrrole heme *d*_1_ plays an important role for bacterial denitrification as essential cofactor of the cytochrome *cd*_1_ nitrite reductase. Structurally, heme *d*_1_ is a dioxo‐isobacteriochlorin carrying two keto functions on pyrrole rings A and B. While most of the steps of heme *d*_1_ biosynthesis were elucidated, the introduction of the keto functions remained enigmatic. In this study, we show that the proteins NirF and NirC catalyze the formation of the keto functions. NirF binds the nonenzymatically formed dilactone form of 3,8‐dideoxo‐dihydro‐heme *d*_1_ and together with NirC transforms this intermediate into dihydro‐heme *d*_1_ as shown by HPLC‐UV/Vis and HPLC‐MS analysis. The additional oxygen atoms of dihydro‐heme *d*_1_ derive from water based on experiments performed with ^18^O‐labeled H_2_O.

## Graphical abstract

During heme *d*_1_ biosynthesis, the proteins NirF and NirC together catalyze the formation of dihydro‐heme *d*_1_, thereby introducing the unique keto functions of heme *d*_1_. In this paper, the development of an in vitro enzyme activity assay for NirF/NirC is described, which demonstrates the function of the two proteins for the first time.

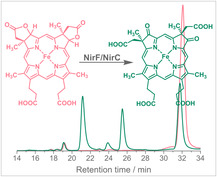

## 1 Introduction

**SCHEME 1:**
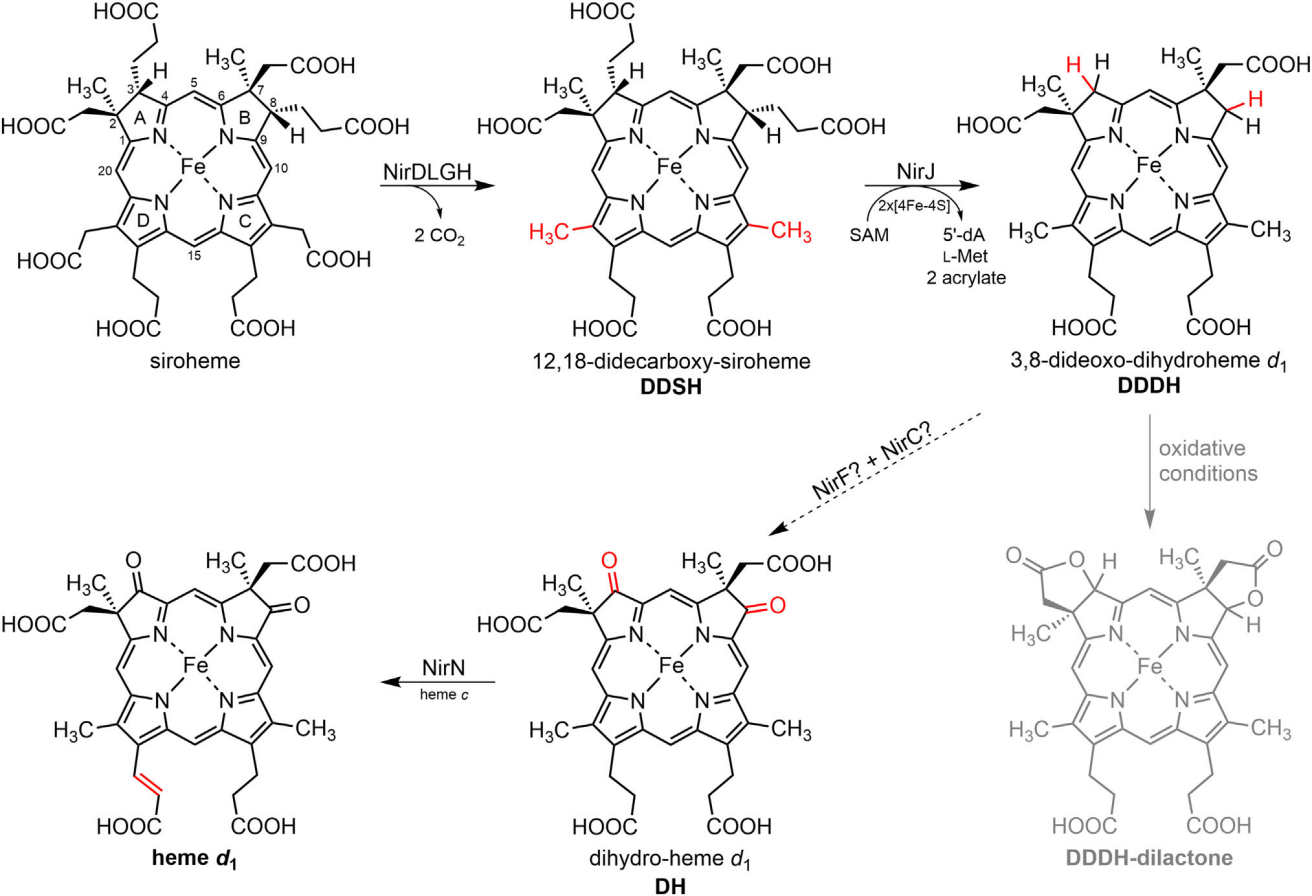
Heme *d*_1_ biosynthesis pathway starting from siroheme. Reaction steps catalyzed by the enzymes NirDLGH, NirJ, and NirN were established in previous studies [5, 6, 7, 8, 9], and the respective structural changes are highlighted in red. Cofactors of NirJ and NirN are given below the arrows. The conversion of 3,8‐dideoxo‐dihydroheme *d*_1_ to dihydro‐heme *d*_1_ is proposed to be catalyzed by NirF and NirC and is the subject of this work. IUPAC numbering of C‐atoms and pyrrole ring designations (A–D) are indicated for siroheme. Abbreviations: 5′‐dA, 5′‐deoxyadenosine; l‐Met, l‐methionine; SAM, *S*‐adenosyl‐l‐methionine.

Heme *d*_1_ is an iron‐containing tetrapyrrole that exhibits a dioxo‐isobacteriochlorin structure [1]. It functions as the catalytically essential cofactor of cytochrome *cd*_1_ nitrite reductases (NirS) [2], which catalyze the second step of denitrification in some denitrifying bacteria such as *Pseudomonas aeruginosa* [3]. The characteristic hallmarks of the heme *d*_1_ structure are the keto functions on pyrrole rings A and B as well as the acrylate side chain on pyrrole ring D (Scheme 1). Biosynthetically, heme *d*_1_ is derived from the common tetrapyrrole precursor 5‐aminolevulinic acid, and its synthesis proceeds via the key intermediates uroporphyrinogen III and siroheme [4, 5]. After siroheme formation, the next steps of heme *d*_1_ biosynthesis are catalyzed by the siroheme decarboxylase NirDLGH, which converts siroheme into 12,18‐didecarboxy‐siroheme (DDSH) [5, 6], and the radical *S*‐adenosyl‐l‐methionine (SAM) enzyme NirJ that cleaves off two propionate side chains on pyrrole rings A and B to form 3,8‐dideoxo‐dihydro‐heme *d*_1_ (DDDH) (Scheme 1) [7, 8]. All these reaction steps take place in the bacterial cytoplasm, since the respective enzymes are all cytoplasmatic. In contrast, the final step of heme *d*_1_ biosynthesis, in which the enzyme NirN catalyzes the conversion of dihydro‐heme *d*_1_ (DH) into heme *d*_1_, as well as the insertion of the cofactor into NirS, takes place in the periplasm [9, 10]. In the description of heme *d*_1_ biosynthesis so far, there is still one uncharacterized step, namely the conversion of the NirJ reaction product DDDH into the last heme *d*_1_ precursor DH (Scheme 1). It was proposed by Klünemann et al. that the proteins NirF and NirC might be responsible for this reaction [11]. Since both, NirF and NirC, are located in the periplasm [10, 12, 13], DDDH would have to cross the inner membrane by a so far unknown transport mechanism. In previous studies, it was shown that NirF is essential for heme *d*_1_ biosynthesis [14, 15, 16] and that it is able to bind heme *d*_1_ as well as DH [11, 12]. In some bacteria, inter alia *P. aeruginosa*, NirF carries a lipid anchor by which it is attached to the periplasmic side of the inner membrane [10, 17]. NirC is a small, *c*‐type cytochrome that was observed to be essential for heme *d*_1_ biosynthesis in some denitrifying bacteria [14]. On the other hand, in *P. aeruginosa*, NirC was not required for heme *d*_1_ biosynthesis, and it was proposed that it might play a role as an electron donor for NirS [13]. In contrast, NirC from *Paracoccus pantotrophus* was observed to act as electron donor for NirN in vitro [18]. For NirF and NirC, both from *P. aeruginosa*, crystal structures were solved [11, 19]. NirF is a single domain protein adopting an eight‐bladed β‐propeller fold that is similar to the heme *d*_1_‐binding domain of NirS and the DH‐binding domain of NirN [2, 20]. The structure of NirF in complex with DH allowed for the identification of amino acid residues involved in tetrapyrrole binding. Two histidine residues, one arginine and one lysine residue, were identified to be essential for the function of NirF in vivo [11]. Similarly, one of the essential histidine residues had been previously identified in NirF from *P. pantotrophus* [12]. The structure of NirC exhibits the characteristic fold for monoheme *c*‐type cytochromes [19]. Despite the studies on NirF and NirC described above, the physiological function of both proteins remained unclear. The latest proposal was that NirF, potentially together with NirC, catalyzes the conversion of the NirJ reaction product into DH, and, in this scenario, the NirF/DH complex structure would represent the enzyme/product complex [11].

The aim of this study was to clarify the roles of NirF and NirC and to elucidate the last missing step of heme *d*_1_ biosynthesis. For this purpose, purified NirF and NirC from *P. aeruginosa* were used to establish an in vitro enzyme activity assay with the NirJ reaction product DDDH as the potential substrate. The formation of DH was observed in the presence of both NirF and NirC. However, the actual substrate that binds to NirF is the dilactone form of DDDH. We also show that the additional oxygen atoms, which are introduced by NirF/NirC, are derived from water. Potential mechanisms for the conversion of DDDH‐dilactone to DH are discussed.

## 2 Experimental Section

### 2.1 Protein Production and Purification

Recombinant NirJ from *D. shibae* (UniProt ID, A8LLZ7; gene locus name, Dshi_3173) and NirF (UniProt ID, Q51480; gene locus name, PA0516) and NirC (UniProt ID, A0A0H2ZKR4; gene locus name, PA14_06730) from *P. aeruginosa* were produced and purified as published previously [8, 11, 19]. For details, see Supporting Information.

## 3 Enzyme Activity Assays

### 3.1 NirJ Reaction

The NirJ reaction was conducted as described previously [8] in order to produce the potential substrate of NirF/NirC. NirJ reaction mixtures (in buffer A: 50 mM HEPES, pH 7.5, 300 mM NaCl, 30% glycerol (w/v)) contained 20–100 µM NirJ/DDSH, 10 equivalents of SAM and sodium dithionite, and 1 mM sodium nitrite, and were incubated at 30°C for 60 min under anaerobic conditions (anaerobic glove box containing 95% N_2_/5% H_2_) in the dark. The total volume of the NirJ reaction mixtures varied depending on the subsequent NirF/NirC assay. Note that the initial concentrations of DDSH might vary between different NirJ/DDSH preparations, and stated concentrations are protein concentrations for NirJ.

## 4 NirF/NirC Reactions

In initial NirF/NirC activity assays, the aerobically purified NirF and NirC were directly added to the NirJ reaction mixture. The NirJ reaction was performed as described above in a total volume of 500 µL and contained 100 µM NirJ/DDSH. After 60 min of incubation, a 250 µL sample was withdrawn and stopped by addition of 5 µL concentrated HCl, and tetrapyrroles were extracted as described in the Supporting Information. The remaining 250 µL of the NirJ reaction was supplemented with NirF and NirC at final concentrations of 20 and 40 µM, respectively, in a total volume of 1.25 mL of buffer A. The mixture was further incubated for 2.5 h at 22°C under anaerobic conditions in the dark. Then, a 250 µL sample was stopped with 5 µL concentrated HCl, and tetrapyrroles were extracted for HPLC analysis as described in the Supporting Information.

For NirF/NirC activity assays under aerobic conditions (laboratory atmosphere), the DDDH‐dilactone was produced by performing the NirJ reaction (40 µM NirJ/DDSH in a volume of 250 µL) and subsequent extraction of the reaction product with acidified ethyl acetate as previously reported [8] and described in the Supporting Information. The dried tetrapyrrole was then dissolved by the addition of purified NirF and NirC in their respective protein buffers (see Supporting Information) at final concentrations of 40 and 80 µM, respectively. The final volumes of these reactions usually ranged from 250 to 350 µL, and the mixtures were incubated for 2.5 h at 22°C in the dark. Then, the reactions were stopped by the addition of 5 µL concentrated HCl to 250 µL of reaction mixture, and tetrapyrroles were extracted for HPLC analysis as described in the Supporting Information.

## 5 NirF Reaction

For the NirF assay (without NirC), the DDDH‐dilactone was produced by performing the NirJ reaction (80 µM NirJ/DDSH in a volume of 250 µL) and subsequent extraction of the reaction product with acidified ethyl acetate. The dried tetrapyrrole was dissolved by addition of purified NirF at a final concentration of 40 µM in 250 µL NirF buffer (10 mM Tris‐HCl, pH 8, 500 mM NaCl, 30% (w/v) glycerol). The mixture was incubated under aerobic conditions for 30 min at 22°C in the dark. The reaction was stopped by the addition of 5 µL concentrated HCl, and tetrapyrroles were extracted for HPLC analysis as described in the Supporting Information.

## 6 NirF/C Reaction with ^18^O‐Water

For the NirF/NirC activity assay with ^18^O‐water (97% ^18^O, Deutero GmbH, Kastellaun, Germany), the NirF and NirC protein buffers were prepared with the labeled H_2_^18^O. Buffer exchange was performed after protein purification for purified NirF and NirC by repeated cycles (6×) of concentrating the protein solutions (by factor two) and subsequent diluting (1:1) with the H_2_^18^O‐buffers. The final protein solutions should contain about 95% H_2_^18^O (by calculation). Then, the aerobic NirF/NirC activity assay was performed as described above.

## 7 NirF Tetrapyrrole Binding Assay

For the NirF tetrapyrrole binding assay, the NirJ reaction was performed as described above in a total volume of 250 µL containing 80 µM NirJ/DDSH. After 60 min of incubation, NirJ was precipitated by heat denaturation (95°C, 5 min) and pelleted by centrifugation (15,000 g, 10 min). Then, the tetrapyrrole containing supernatant was split in two 100‐µL aliquots. One aliquot was exposed to air (laboratory atmosphere) in order to convert the DDDH to the dilactone form, while the second aliquot was kept under anaerobic conditions. UV/Vis absorption spectra for both tetrapyrrole solutions were recorded under anaerobic conditions. Then, 100 µL NirF (40 µM final concentration) were added to each tetrapyrrole solution under anaerobic conditions, and the mixtures were incubated for 30 min, after which UV/Vis absorption spectra were again recorded.

## 8 High‐Performance Liquid Chromatography

**FIGURE 1:**
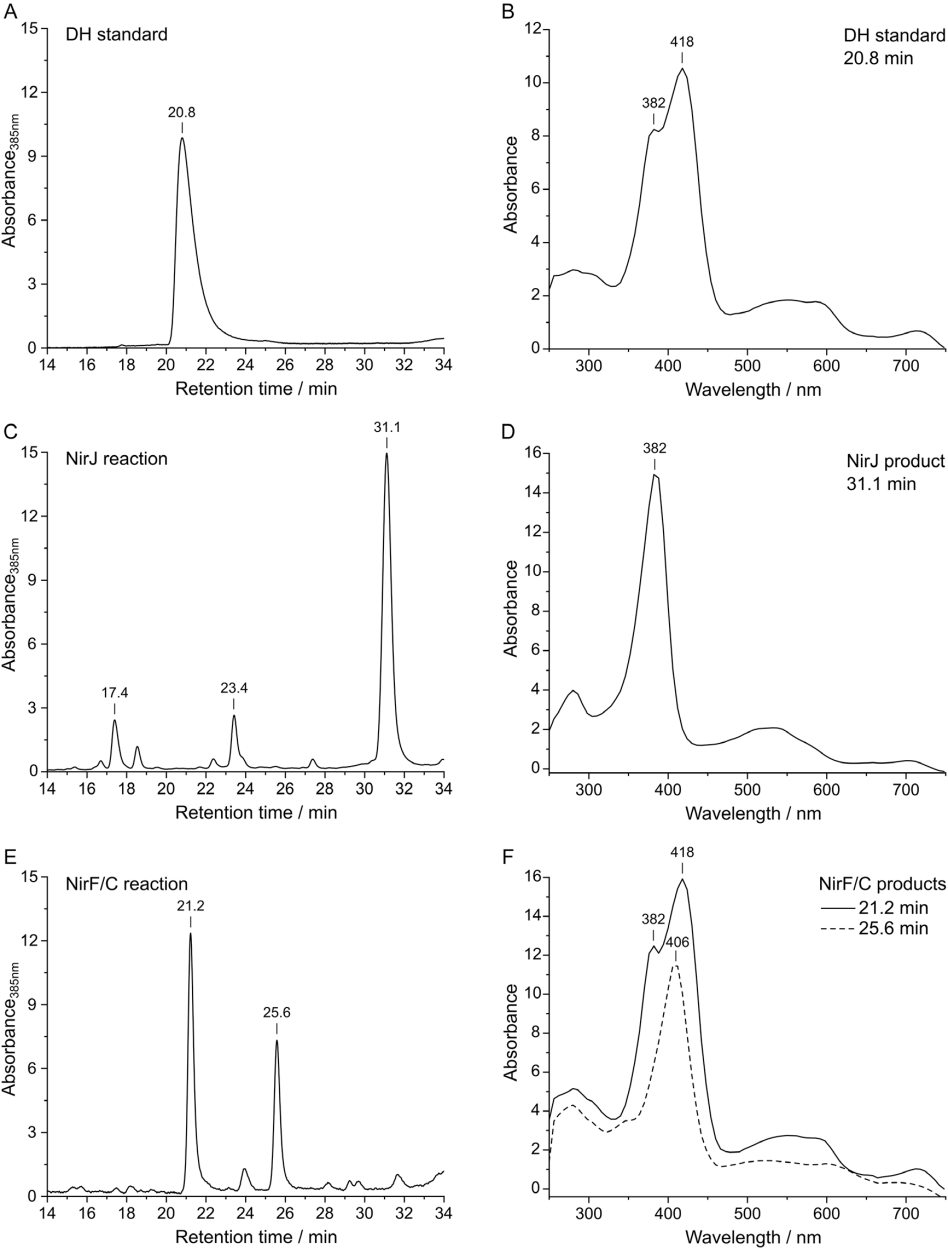
HPLC‐UV/Vis analysis of the initial NirF/NirC activity assay. HPLC chromatograms of the dihydro‐heme *d*_1_ standard (A), the NirJ reaction mixture (C), and the NirF/NirC reaction mixture (E) were recorded by measuring the absorbance at 385 nm. Relevant peaks are labeled with the corresponding retention times, respectively. UV/Vis absorption spectra at the indicated retention times are shown for the dihydro‐heme *d*_1_ standard (B), the NirJ reaction product (D), and the NirF/NirC reaction products (F). Main absorption maxima are labeled with the respective wavelength. The initial NirJ reaction mixture contained 100 µM NirJ and was diluted by a factor of five due to the addition of NirF (20 µM) and NirC (40 µM) for the NirF/NirC reaction. Therefore, the chromatogram in (C) and UV/Vis spectrum in (D) were normalized by division of the original data by five. The chromatograms and UV/Vis spectra are representative for three replicates with different enzyme preparations.

**FIGURE 2:**
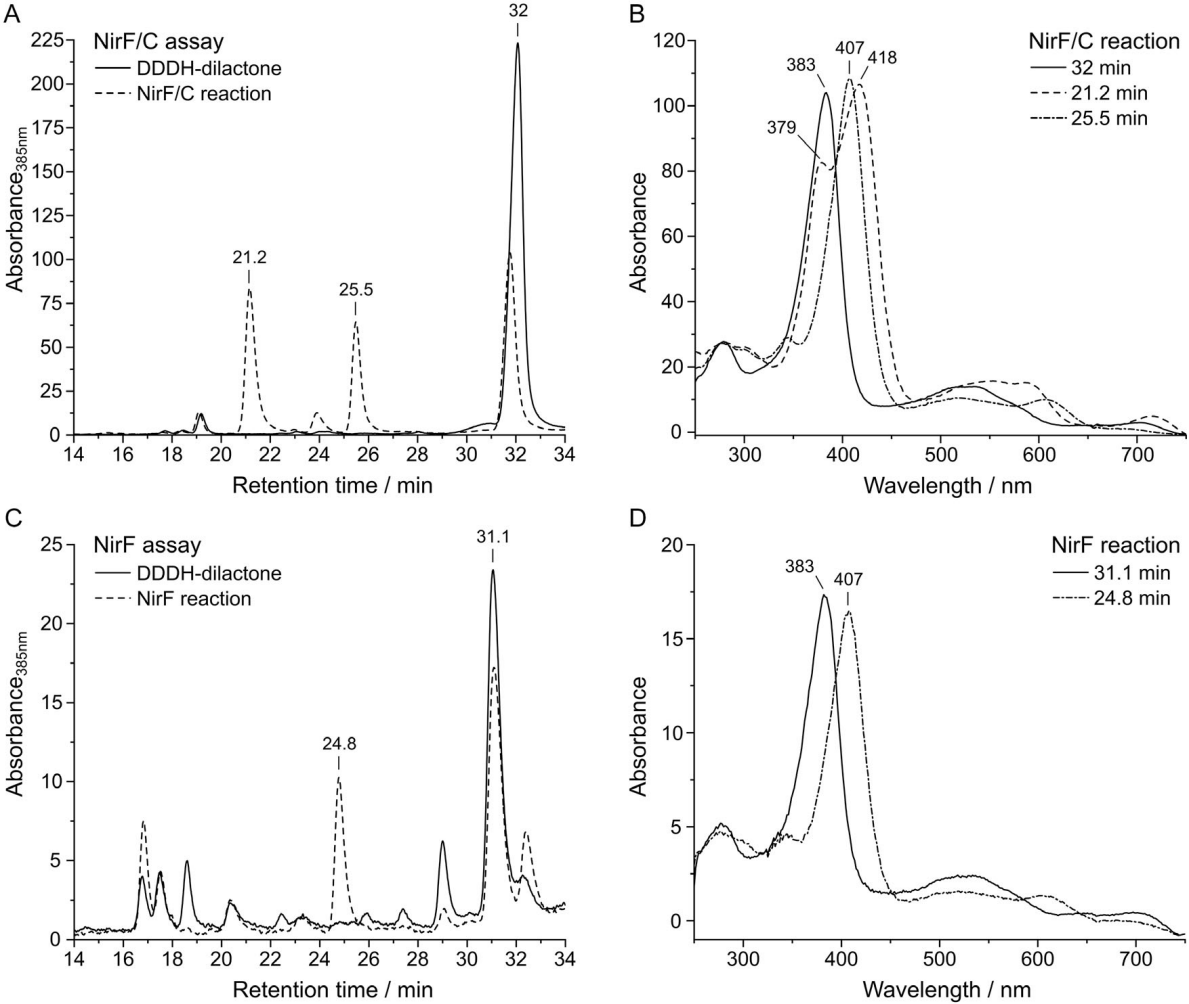
HPLC‐UV/Vis analysis of the aerobic NirF/NirC and NirF activity assays with the extracted DDDH‐dilactone as substrate. (A) HPLC chromatograms of the DDDH‐dilactone (extracted NirJ reaction product) and the NirF/NirC reaction products. (B) UV/Vis absorption spectra for the NirF/NirC reaction products and the substrate at the indicated retention times. (C) HPLC chromatograms of the DDDH‐dilactone and the NirF reaction products. (D) UV/Vis absorption spectra for the main NirF reaction product and the substrate at the indicated retention times. HPLC chromatograms were recorded by measuring the absorbance at 385 nm, and relevant peaks are labeled with the corresponding retention times, respectively. In the UV/Vis spectra, main absorption maxima are labeled with the respective wavelength. The chromatograms and UV/Vis spectra are representative for three replicates with different enzyme preparations.

HPLC analysis of NirJ and NirF/NirC reaction products with UV/Visible detection were performed on a JASCO HPLC 2000 series system (Jasco, Gross‐Umstadt, Germany) as described previously [8] and in the Supporting Information. For UV/Vis spectra, data intervals were either 6 nm (Figure 1) or 1.5 nm (Figure 2).

HPLC‐MS analysis was performed on a Thermo Fisher Scientific HPLC UltiMate 3000 System TSQ Quantum Access Max mass spectrometer. HPLC was conducted on a Reprosil‐Pur C18‐AQ column (Dr. Maisch HPLC GmbH, Ammerbuch‐Entringen, Germany) as described before [8] with modifications of the solvent gradient: Start condition was 80% solvent A (0.1% formic acid in water) and 20% solvent B (acetonitrile). After sample injection, the start condition was held for 15 min, after which the concentration of B was increased to 100% within 5 min. 100% B was held for 3 min before returning to the start condition within 2 min. Mass spectra were recorded in positive ion mode with instrument settings identical to those reported previously [8] and given in the Supporting Information.

## 9 Results and Discussion

### 9.1 NirF and NirC Catalyze the Formation of Dihydro‐Heme *d*_1_

In order to test the hypothesis that NirF and NirC catalyze the conversion of the NirJ reaction product to DH, an initial enzyme activity assay was performed using purified, recombinant NirF and NirC from *P. aeruginosa* [11, 19] and the purified NirJ/DDSH complex from *Dinoroseobacter shibae* [8] (Figure S1). In the first step of the assay, the NirJ reaction (100 µM NirJ/DDSH, 10 equivalents of SAM and sodium dithionite, 1 mM sodium nitrite) was allowed to proceed for 60 min under anaerobic conditions in order to produce the potential substrate for NirF/NirC. After the 60 min incubation, an aliquot of the mixture was withdrawn for tetrapyrrole extraction and high‐performance liquid chromatography (HPLC) analysis. Next, NirF (20 µM) and NirC (40 µM) were directly added to the remaining NirJ reaction, and the assay mixture was further incubated for 2.5 h. For both samples, before and after incubation with NirF/NirC, the tetrapyrroles were extracted using ethyl acetate and analyzed by HPLC with UV/Visible detection (HPLC‐UV/Vis). For comparison, a DH standard was also analyzed (Figure 1).

The HPLC chromatogram of the sample that was withdrawn before the addition of NirF/NirC (Figure 1C) exhibited one major peak at a retention time of about 31 min representing the NirJ reaction product DDDH in its dilactone form obtained after aerobic extraction [7, 8]. The corresponding UV/Vis absorption spectrum at 31.1 min exhibited a maximum at 382 nm and a broad absorption feature around 532 nm (Figure 1D). The two minor peaks in the chromatogram at retention times of 17.4 and 23.4 min correspond to the NirJ substrate DDSH and the NirJ reaction intermediate with only one propionate side chain removed, respectively, as previously identified by HPLC‐MS [7]. In the chromatogram of the sample that was taken after incubation with NirF/NirC, the peak at 31 min had almost completely disappeared and two new peaks at about 21 min and 25.6 min were visible indicating the conversion of the NirJ reaction product into new substances (Figure 1E). The UV/Vis absorption spectrum of the new substance at 21.2 min retention time exhibited maxima at 382 and 418 nm and a broad feature between 500 and 620 nm (Figure 1F), which is significantly different compared to the spectrum of the DDDH‐dilactone. The HPLC analysis of a DH standard (Figure 1A,B) revealed a retention time of about 21 min and a UV/Vis absorption spectrum with identical maxima compared to that of the NirF/NirC product at 21 min. Based on the identical retention times and absorption spectra of the NirF/NirC product and the DH standard, we concluded that NirF and NirC indeed catalyze the formation of DH. The absorption spectrum corresponding to the second peak at 25.6 min in the HPLC chromatogram of the NirF/NirC reaction displayed a maximum at 406 nm and a broad feature between 470 and 650 nm (Figure 1F). This new product could represent an intermediate of the NirF/NirC reaction (see below).

### 9.2 The NirF/NirC Substrate Is the Dilactone Form of 3,8‐Dideoxo‐Dihydro‐Heme *d*_1_

As described in the foregoing paragraph, the formation of DH was observed after addition of NirF and NirC to a NirJ reaction mixture. Although the assay was performed under anaerobic conditions, both proteins, NirF and NirC, had been purified aerobically and were used in the assay without buffer exchange. Therefore, the final assay mixture was not strictly anaerobic. In the course of several repetitions of the assay, we observed that the activity of NirF/NirC became less the longer the proteins remained in the anaerobic chamber. As one possible cause for this phenomenon, we suspected that the extent of anaerobicity might play an important role for the NirF/NirC reaction. The actual NirJ product DDDH exhibits methylene groups at tetrapyrrole positions C3 and C8, which remain stable in this form under strictly anaerobic conditions. However, under oxidative conditions, for example, upon exposure to air, the acetate groups at positions C2 and C7 undergo lactone formation with C3 and C8 (Scheme 1) [7, 8]. Therefore, we hypothesized that in the assays with freshly prepared NirF and NirC, the addition of the “aerobic” protein solutions might have caused the formation of the DDDH‐dilactone, which served as the actual substrate for NirF/NirC. However, the longer the protein solutions remained in the anaerobic chamber, the more anaerobic they became. Under these conditions, lactone formation would not take place resulting in the lack of the NirF/NirC substrate.

In order to test this hypothesis, a NirF/NirC activity assay was performed, in which the DDDH‐dilactone was produced by purpose. For this, the anaerobic NirJ reaction product DDDH was aerobically extracted with ethyl acetate, conditions under which the DDDH‐dilactone is formed [7, 8]. The extracted and dried tetrapyrrole was redissolved in buffer containing NirF and NirC, and the mixture was incubated under aerobic conditions for 2.5 h. Then, the extracted reaction products as well as the DDDH‐dilactone obtained after the NirJ reaction were analyzed by HPLC‐UV/Vis (Figure 2).

As expected, the chromatogram of the aerobically extracted DDDH exhibited one major peak at a retention time of 32 min, and the corresponding absorption spectrum matched that of the DDDH‐dilactone (Figure 1D) with the main absorption maximum at 383 nm. In the chromatogram of the NirF/NirC reaction mixture, the DDDH‐dilactone peak at 32 min was reduced and two peaks at about 21 min and 25.5 min appeared representing the NirF/NirC reaction product and potential intermediate. The retention time and UV/Vis absorption spectrum of the product at 21.2 min were highly similar to the DH standard (Figure 1B) with main maxima at 379 and 418 nm. Therefore, NirF/NirC accepted the DDDH‐dilactone as substrate and converted it into DH. For the peak at 25.5 min, the corresponding UV/Vis absorption spectrum displayed a major maximum at 407 nm and broader features at about 520 and 607 nm, highly similar to the spectrum of the potential reaction intermediate shown in Figure 1F. Interestingly, when the aerobic activity assay was performed with only NirF and incubated for 30 min, the same peak at about 25 min was also observed (Figure 2C, D). Apparently, the formation of the potential intermediate did not require the presence of NirC. In contrast, efficient formation of DH was observed only in the presence of both NirF and NirC.

**FIGURE 3:**
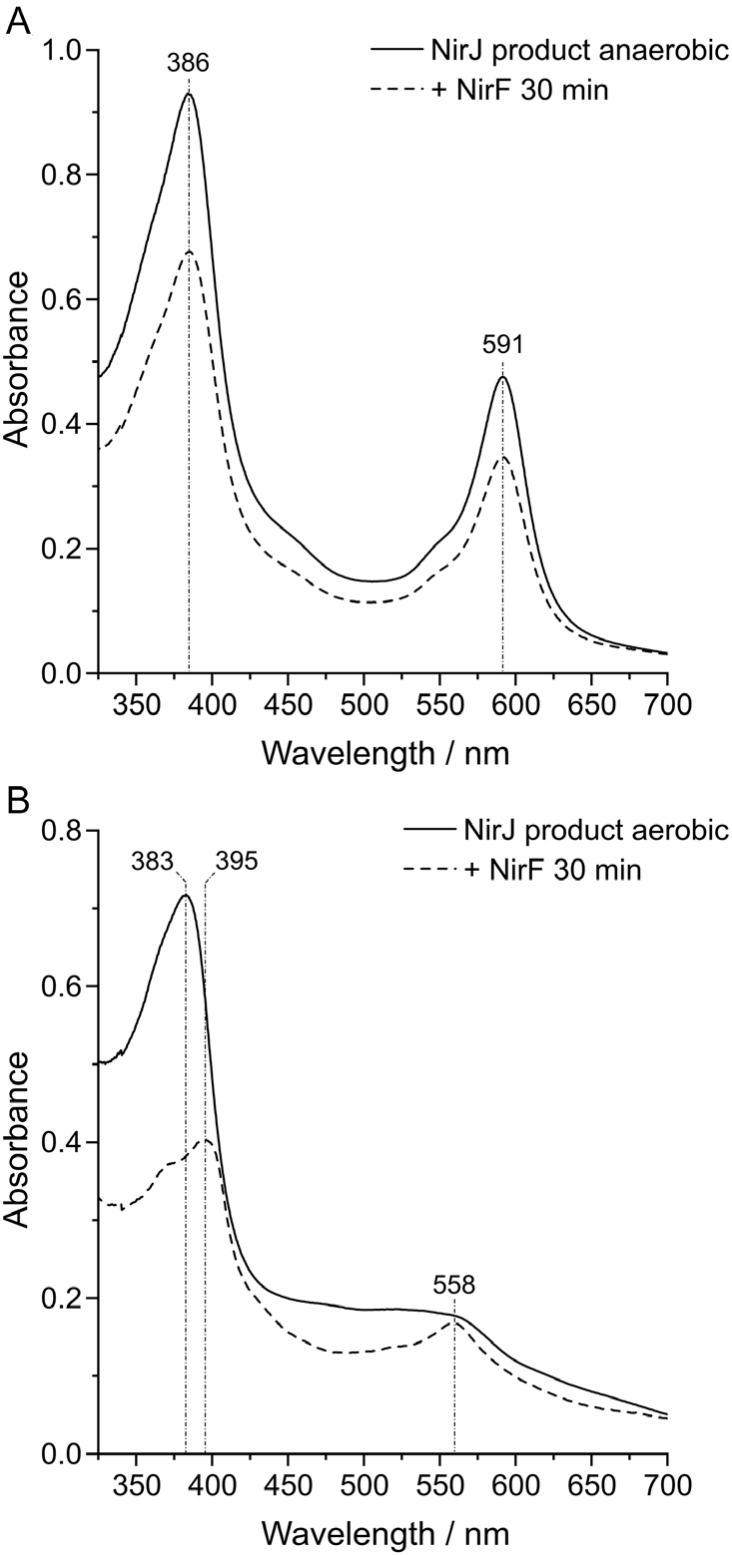
UV/Vis absorption spectra of tetrapyrrole binding assays with NirF. (A) UV/Vis absorption spectra of the anaerobic NirJ product (DDDH) before and after NirF addition. (B) UV/Vis absorption spectra of the aerobic NirJ product (DDDH‐dilactone) before and after NirF addition. Main absorption maxima are labeled with the respective wavelength. The spectra of the anaerobic and aerobic NirJ product were normalized by division of the original data by two in order to compensate for the dilution due to NirF addition. The UV/Vis spectra are representative for two replicates with different enzyme preparations.

The finding that NirF/NirC accept the DDDH‐dilactone as substrate was not expected. In order to further clarify whether both DDDH and DDDH‐dilactone or solely the dilactone serve as substrates, a tetrapyrrole binding assay was performed with NirF, which was assumed to be the substrate binding protein. For this purpose, DDDH was produced by the standard NirJ activity assay and then anaerobically extracted by heat denaturation of the protein. While one half of the DDDH preparation was kept strictly anaerobic, the other half was exposed to air in order to form the DDDH‐dilactone. While the anaerobic DDDH solution exhibited a bluish color, the aerobic DDDH‐dilactone solution turned red as previously described [8]. The corresponding UV/Vis absorption spectra exhibit distinct maxima at 386 and 591 nm for DDDH and 383 nm for the DDDH‐dilactone (Figure 3).

In order to test which tetrapyrrole binds to NirF, the protein was added to the respective tetrapyrrole solutions. If binding occurs, a shift of the Soret band around 380 nm and/or a change of the Q‐bands in the 500–600 nm region would be expected. After a 30‐min incubation, UV/Vis absorption spectra of the enzyme/tetrapyrrole mixtures were recorded (Figure 3). For the NirF/DDDH mixture, the wavelengths of the maxima at 386 and 591 nm did not change. In contrast, the addition of NirF to the DDDH‐dilactone solution resulted in a shift of the main maximum from 383 to 395 nm. Additionally, an absorption feature at 558 nm appeared. These results indicated that the DDDH‐dilactone binds to NirF, while DDDH is not accepted by the protein.

**FIGURE 4:**
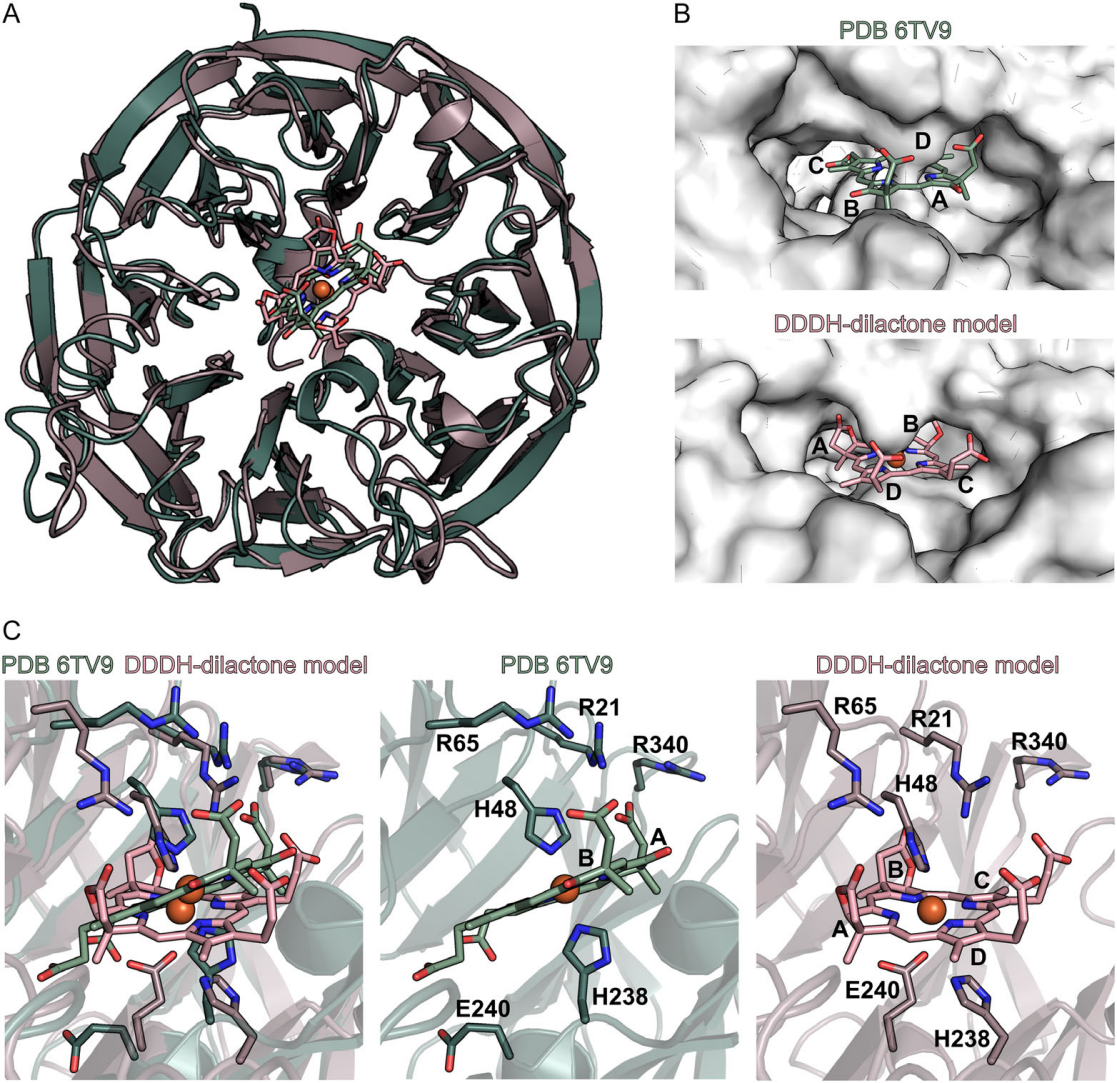
Crystal structure of the NirF/DH complex (PDB 6TV9) [11] and computational model (Chai‐1) [21] of a NirF/DDDH–dilactone complex. (A) Superposition of the crystal structure (green) and the computational model (mauve). NirF is shown as cartoon and the tetrapyrrole ligands as sticks. (B) Surface representation of NirF (light gray) with view onto the tetrapyrrole binding site. DH (upper panel) is shown as green sticks, and the DDDH‐dilactone is represented as rose sticks. The orientation of the pyrrole rings A, B, C, and D is indicated. (C) Detailed views into the tetrapyrrole binding site of NirF: left, superposition of the crystal structure (green) and the computational model (mauve); middle, crystal structure; right, computational model. NirF is shown as cartoon, tetrapyrroles as sticks with pyrrole rings labeled, and coordinating amino acid residues are shown as sticks with labels.

In order to further test the feasibility of DDDH‐dilactone binding to NirF, we performed a computational structure prediction with Chai‐1 [21] for NirF and the DDDH‐dilactone as ligand (Figure 4). Overall, the predicted structure is highly similar to the experimental crystal structure. Also, the overall binding site of the DDDH‐dilactone is identical to that of DH in the crystal structure including the same coordinating amino acid residues. However, while in the crystal structure the C3 (pyrrole ring A) and C8 (pyrrole ring B) positions are oriented toward the opening of the putative substrate binding site, these positions point toward the interior of the potential active site pocket in the computational model. Although computational models should be interpreted with caution, the structural model of the NirF/DDDH–dilactone complex at least shows that the dilactone can be accommodated within the active site of NirF supporting our finding that the DDDH‐dilactone is accepted as substrate by the enzyme.

**SCHEME 2:**
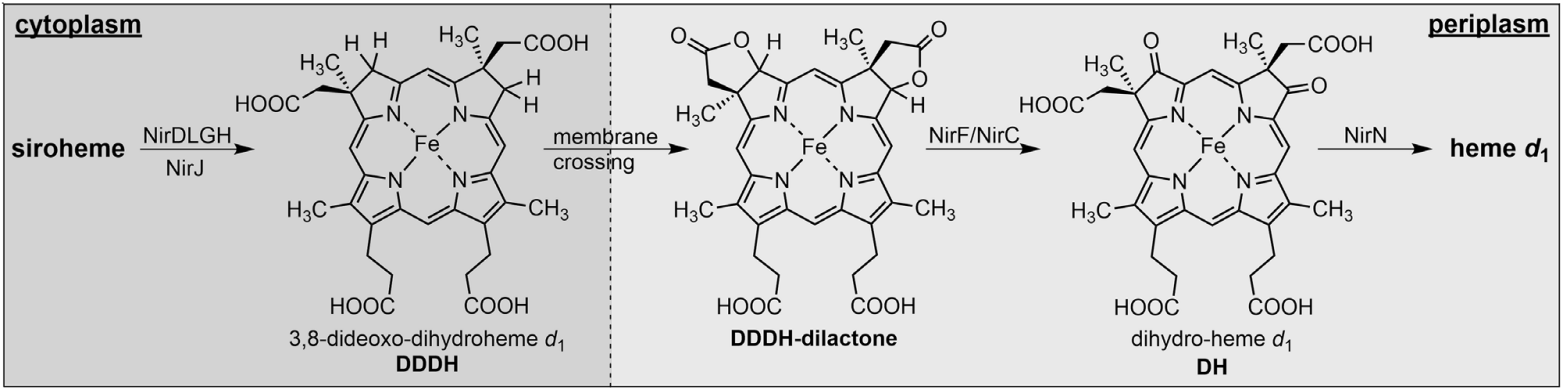
Cellular localization of heme *d*_1_ biosynthesis steps in gram‐negative bacteria. While all enzymatic steps up to the formation of the intermediate DDDH take place in the bacterial cytoplasm (darker gray, left side), the remaining steps are catalyzed by NirF/NirC and NirN in the periplasm (lighter gray, right side). DDDH must cross the cytoplasmic membrane and be converted to the dilactone. The mechanisms of both membrane crossing and dilactone formation are not known yet.

Based on all experiments described above, we conclude that the formation of DH catalyzed by NirF/NirC requires the conversion of the NirJ reaction product DDDH to the DDDH‐dilactone, which serves as the actual substrate for NirF. NirF binds and converts the DDDH‐dilactone into a so far uncharacterized intermediate (retention time 25 min), while in the presence of both NirF and NirC, DH is formed in addition to the intermediate. In our in vitro enzyme activity assays, the DDDH‐dilactone was produced by exposing DDDH to air. In vivo, such a scenario is less likely, since denitrification usually takes place under anaerobic conditions. However, in this context, the different cellular locations of the heme *d*_1_ biosynthesis steps according to the enzyme localizations must be considered (Scheme 2). All steps up to DDDH formation take place in the cytoplasm as they are catalyzed by cytoplasmic enzymes including NirJ. In contrast, NirF, NirC, and NirN are located in the periplasm [10, 12, 18]. Therefore, DDDH produced by NirJ must cross the cytoplasmic membrane by a so far unknown mechanism. Conversion of DDDH to the dilactone might occur either during transport via a specific transporter or after release into the periplasmic space due to the more oxidizing environment of the periplasm compared to the cytoplasm. Although lactone formation of isobacteriochlorins was described previously [22, 23], the exact mechanism of this intramolecular reaction is not known. For heme and coproheme, in vitro lactone formation and hydroxylation reactions of a pyrrole ring were reported, which required the hydrogen peroxide–mediated formation of an oxidized compound I intermediate [24, 25]. However, in our case, a hydrogen peroxide–mediated reaction is not very likely.

### 9.3 The Oxygen Atoms Introduced by NirF/NirC Are Derived from Water

**FIGURE 5:**
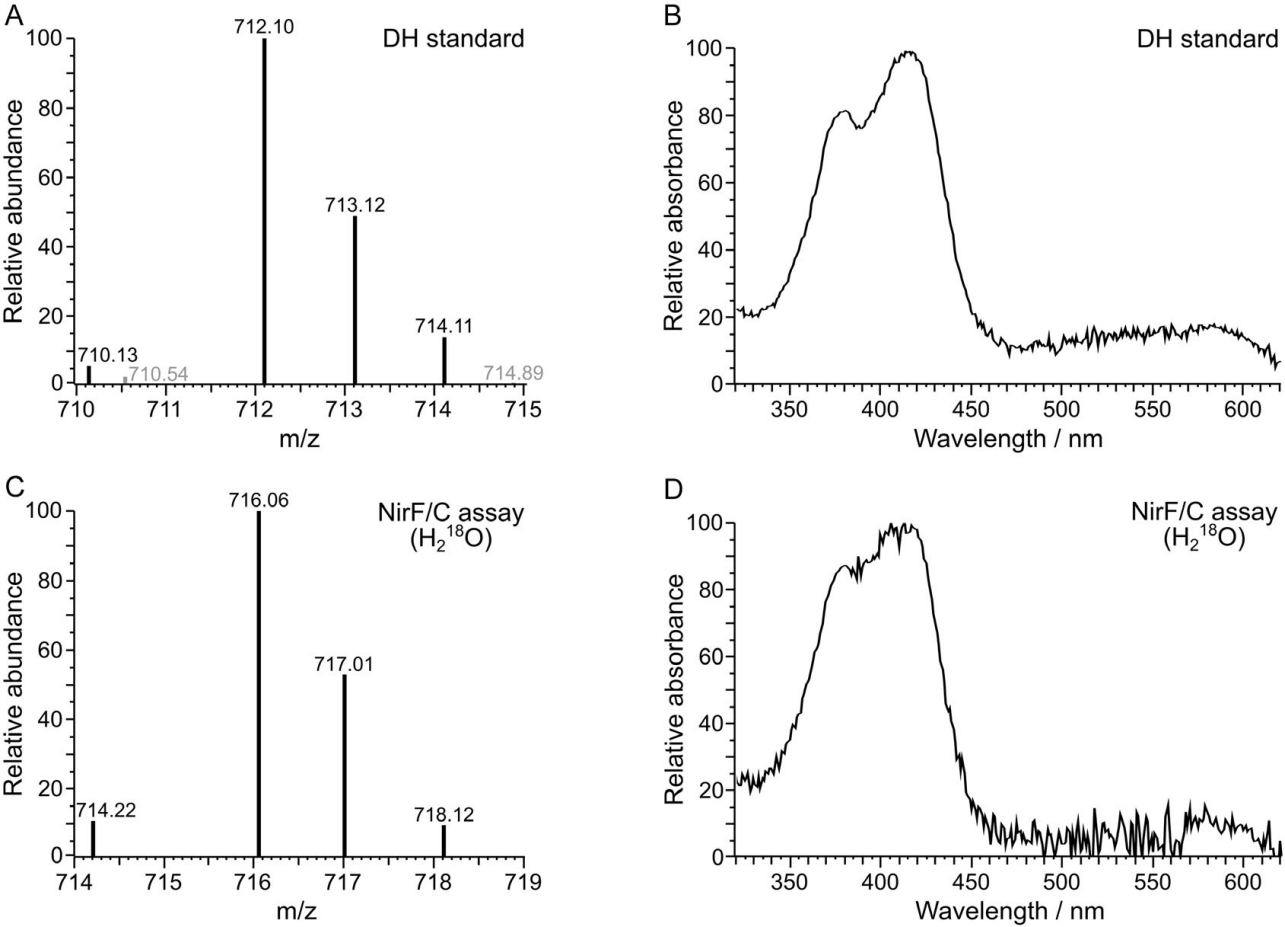
Mass spectrometric analysis (positive ion mode) of the aerobic NirF/NirC assay in H_2_^18^O‐containing buffer. (A) Mass spectrum of the dihydro‐heme *d*_1_ (DH) standard (black, calculated isotopic pattern (m/z): 712.15 (100.0%), 713.15 (39.8%), 714.15 (10.5%), 710.15 (6.4%), 711.15 (2.4%), 713.14 (1.5%), 715.15 (1.2%)). Mass signals not belonging to DH are shown in gray. (B) UV/Vis absorption spectrum of the DH standard. (C) Mass spectrum of DH produced by NirF/NirC in H_2_^18^O‐containing buffer (calculated isotopic pattern for ^18^O‐labeled DH (m/z): 716.15 (100.0%), 717.16 (39.8%), 718.16 (9.9%), 714.16 (6.4%), 715.16 (2.5%), 719.16 (1.8%), 717.15 (1.5%)). (D) UV/Vis absorption spectrum of the DH produced by NirF/NirC in H_2_^18^O‐containing buffer. Spectra shown in (C) and (D) represent a single experiment.

In the course of the characterization of NirF/NirC as DH synthase, we also addressed the question of the origin of the oxygen atoms that are introduced during the conversion of DDDH‐dilactone into DH. Considering that heme *d*_1_ formation is associated with anaerobic denitrification, the most likely hypothesis was that the oxygen atoms are derived from water and not from molecular oxygen. In order to test this hypothesis, the NirF/NirC activity assay with the DDDH‐dilactone substrate was performed in buffer containing about 95% ^18^O‐labeled water. After tetrapyrrole extraction, HPLC coupled to mass spectrometry (HPLC‐MS) analysis was conducted to determine the mass of the formed DH. For comparison, a DH standard was analyzed under identical conditions. As shown in Figure 5, an *m/z* = 712.10 was observed for the DH standard in good agreement with the calculated mass of DH (*m/z* = 712.15). Moreover, the isotopic pattern of the experimental mass spectrum of the DH standard matched that of the calculated spectrum, and the UV/Vis absorption spectrum of the standard showed the expected absorption maxima at about 380 and 416 nm. When the extracted product of the NirF/NirC reaction in ^18^O‐water‐containing buffer was analyzed by HPLC‐MS, an *m/z* = 716.06 was observed. The identity of the detected product as DH was confirmed by the isotopic pattern of the mass spectrum as well as the corresponding UV/Vis absorption spectrum (Figure 5). The observed mass difference of + 4 Da compared to the mass of the unlabeled DH standard suggested the incorporation of two ^18^O atoms derived from the heavy water during the NirF/NirC reaction.

### 9.4 Hypothetical Mechanisms for the Formation of Dihydro‐Heme *d*_1_

**SCHEME 3:**
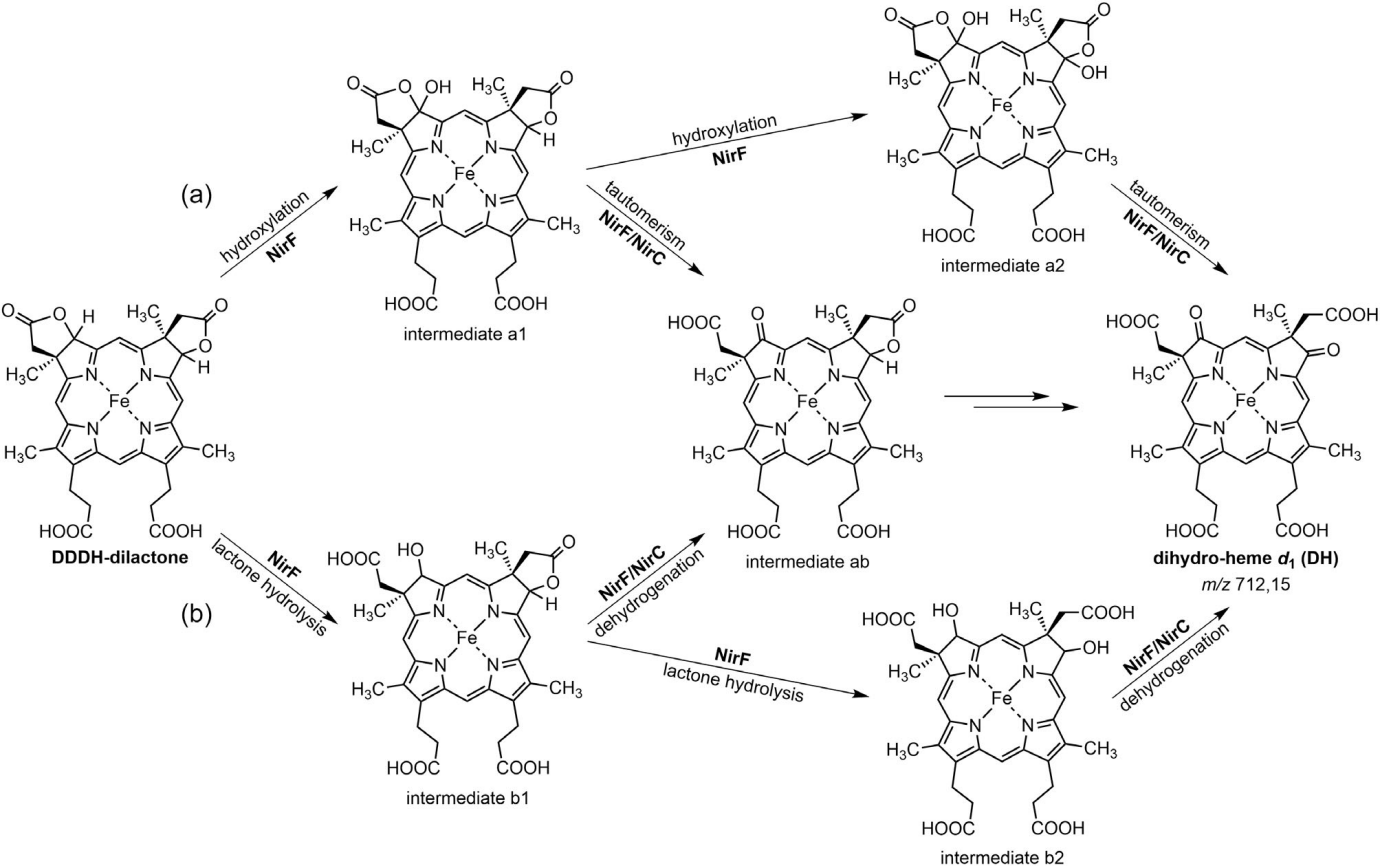
Potential mechanisms for the formation of dihydro‐heme *d*_1_ catalyzed by NirF/NirC. NirF could act either as a hydroxylase (a) or as an esterase (b). In scenario (a), NirC potentially assists in release of intermediates enabling the nonenzymatic tautomerization reaction. In (b), NirC might act as an electron acceptor for the dehydrogenation reaction.

Based on the experimental results described in this study, at least two hypothetical mechanisms for the introduction of the keto functions of DH catalyzed by NirF/NirC are plausible (Scheme 3). Both proposals consist of two steps, and in both scenarios, the first step is catalyzed by NirF, while the second step requires the additional assistance of NirC. In the first mechanistic proposal (a), NirF catalyzes the hydroxylation of DDDH‐dilactone at position C3 (or C8) to yield an intermediate a1. This intermediate could undergo tautomeric rearrangement upon release from the enzyme, a process that might require the presence of NirC. As a result, one of the keto functions is formed. The repetition of this reaction sequence at the second position (C8 or C3) would yield the reaction product DH. In this scenario, the oxygen atoms derived from water would yield the keto functions. Alternatively, in the second mechanistic proposal (b), NirF acts as an esterase catalyzing the hydrolysis of the lactone, which results in the formation of an intermediate b1. The conversion of the hydroxyl group at C3 (or C8) into the keto function represents a dehydrogenation, for which NirC might serve as an electron acceptor. Again, repetition of these reaction steps would yield DH. In this case, the water‐derived O‐atoms would reside in the carboxylate groups. As an alternative to the proposed stepwise introduction of the keto functions, it is also thinkable that hydroxylation or lactone hydrolysis first takes place at both positions, C3 and C8, before the keto functions are formed. In any case, further experiments are required in order to elucidate the mechanism of DH formation and to clarify the individual roles of NirF and NirC in the overall reaction. A crucial step toward this goal will be the identification the potential reaction intermediate(s).

## 10 Conclusion

In this study, we show that NirF and NirC together catalyze the formation of dihydro‐heme *d*_1_, the penultimate step of heme *d*_1_ biosynthesis, which was unknown so far. Based on our experimental results, we propose that the actual substrate for NirF/NirC is the dilactone form of the NirJ reaction product DDDH. The exact reaction mechanism, by which the DDDH‐dilactone is converted into DH by NirF/NirC, remains to be determined in future experiments. Also, the transport of DDDH across the cytoplasmic membrane and formation of the dilactone require further investigation. Nevertheless, with this study, the assignment of functions to the various Nir proteins involved in heme *d*_1_ biosynthesis is complete.

## Supporting Information

Additional supporting information can be found online in the Supporting Information section.

## Supporting information

Supplementary Material

## Data Availability

The data that support the findings of this study are available from the corresponding author upon reasonable request.
