# Supplementary material for: The Keto Functions of Heme d 1 Are Introduced by NirF and NirC
Source: Chembiochem. 2026 May 13;27(10):e70370. doi: 10.1002/cbic.70370 (PMC13168839; doi:10.1002/cbic.70370)
Supplement: Supplementary file 1 — Supplementary Material [file CBIC-27-e70370-s001.pdf]

Supporting Information

The Keto Functions of Heme d_1 Are Introduced by NirF and NirC

Pia Hebecker[a] and Gunhild Layer*[a]

[a] Dr. P. Hebecker, Prof. Dr. G. Layer
Institut für Pharmazeutische Wissenschaften, Pharmazeutische Biologie
Albert-Ludwigs-Universität Freiburg
Stefan-Meier-Str. 19, 79104 Freiburg (Germany)
E-mail: gunhild.layer@pharmazie.uni-freiburg.de

Table of contents:

General materials and methods 2
- Plasmids 2
- Protein production and purification 2
- Ethyl acetate extraction of tetrapyrroles 3
- HPLC-UV/Vis analysis 4
- HPLC-MS analysis 4
- Structure prediction with Chai-1 4

Figure S1 5

General materials and methods

Plasmids

The following plasmids were used in this study:

Plasmid	Reference
pACYC_*nirDLGH_nirJ*-His	Boss et al. (2017) *FEBS J.*, 284, 4314.
pKK_*cysG* (pAR8414)	Raux et al. (1996) *J. Bacteriol.*, 178, 753.
pET-SUMO-*nirF*	Klünemann et al. (2021) *FEBS J.*, 288, 244.
pVP008_ompA_*nirC*	Klünemann et al. (2020) *Acta Cryst.*, D76, 375.
pEC86	Arslan et al. (1998) *Biochem. Biophys. Res. Commun.*, 251, 196.

Protein production and purification

NirJ from *D. shibae*

NirJ was produced together with its substrate DDSH and the NirJ/DDSH-complex was purified as described in Meyer et al. (2025), FEBS J., 292, 5151, with minor modifications. Briefly, *E. coli* BL21(DE3) containing the plasmids pKK_*cysG* and pACYC_*nirDLGH_nirJ*-His was used as the production host. Cells were grown at 37 °C in auto-inducing medium ZYM5052 (Studier (2005) Protein Expr. Purif., 41, 207) containing 100 µg/mL ampicillin and 34 µg/mL chloramphenicol until an optical density (600 nm) of 0.6 was reached. Then, the temperature was lowered to 17 °C and the cultures were further cultivated overnight for 15-18 h. The cells were collected by centrifugation and the cell pellet was transferred to an anaerobic chamber containing 95% N_2 / 5% H_2 (Coy Laboratories). All following steps were conducted in the anaerobic chamber using anaerobic buffers. The cells were suspended 1:3 (w/v) in anaerobic buffer A (50 mM HEPES, pH 7.5, 300 mM NaCl, 30 % glycerol (w/v)) supplemented with 25 mM imidazole. The cells were lysed by passage through a French Pressure cell at 1200psi. The soluble protein fraction was obtained by centrifugation at 125,000 *g* for 1 h at 10 °C. The NirJ/DDSH-complex was purified by immobilized metal ion affinity chromatography (IMAC) using a 1.5 mL gravity flow Ni-nitrilotriacetic acid agarose (Macherey-Nagel) column equilibrated with buffer A. After loading the soluble protein fraction onto the column, the column was washed with 4-6 column volumes (CV) of buffer A containing 50 mM imidazole, followed by a second wash step with 2 CV of buffer A containing 75 mM imidazole. The NirJ/DDSH-complex was eluted with buffer A containing 250 mM imidazole. Finally, the imidazole was removed from the protein solution by using a PD-10 desalting column (Cytiva) and buffer A as the final protein buffer. Protein concentrations were determined by the Bradford method with bovine serum albumin as the standard (Bio-Rad Laboratories).

NirF from *P. aeruginosa*

NirF was produced and purified as described in Klünemann et al. (2021) *FEBS J.*, 288, 244, with minor modifications. Briefly, NirF was produced in *E. coli* BL21(DE3) containing the plasmid pET-SUMO-*nirF*. Cells were grown in LB-medium containing 100 µg/mL ampicillin at 37 °C until an optical density (600 nm) of 0.8 was reached. Then, protein production was induced by the addition of 1 mM IPTG (final concentration) and the cultures were further grown overnight for 15-18 h at 20 °C. The cells were collected by centrifugation. The cell pellet was suspended 1:3 (w/v) in buffer B (10 mM Tris-HCl, pH 8, 500 mM NaCl, 10% (w/v) glycerol) containing 10 mM imidazole, and the cells were lysed by passage through a French Pressure

cell at 1200psi. The soluble protein fraction was obtained by centrifugation at 125,000 *g* for 1 h at 18 °C. NirF was purified at room temperature by immobilized metal ion affinity chromatography (IMAC) using a 2.3 mL His60 Ni Superflow column (TaKaRa Bio Inc.) attached to an Äkta pure system (GE Healthcare). The column was equilibrated at a flow rate of 1 mL/min with 5 CV of lysis buffer. After loading the soluble protein fraction on the column, the unbound proteins were washed off with 5 CV lysis buffer. NirF was eluted from the column by applying a linear gradient with increasing imidazole concentration (10-250 mM imidazole) in buffer B. NirF containing fractions were combined, and the buffer was exchanged against NirF buffer (10 mM Tris-HCl, pH 8, 500 mM NaCl, 10% (w/v) glycerol) containing 1 mM DTT by using a PD-10 desalting column (Cytiva). The SUMO tag was cleaved off by the addition of SUMO protease (Sigma Aldrich) (0.01 mg protease per mg NirF) to the NirF solution and incubation overnight. NirF was further purified and freed from the protease by size exclusion chromatography on a HiLoad 16/600 Superdex 200 prep grade column (GE Healthcare) attached to an Äkta pure system. The column was equilibrated at a flow rate of 1 mL/min with 2 CV NirF buffer. 5 mL of protein solution were loaded on the column and the proteins were eluted at a flow rate of 1 mL with NirF buffer. NirF containing fractions were combined. Protein concentrations were determined by the Bradford method with bovine serum albumin as the standard (Bio-Rad Laboratories).

<u>NirC from P. aeruginosa</u>

NirC was produced and purified as described in Klünemann et al. (2020) *Acta Cryst*., D76, 375, with minor modifications. NirC was produced in *E. coli* BL21(DE3) star containing the plasmids pEC86 and pVP008_ompA_*nirC*. Cells were grown in LB-medium containing 30 µg/mL kanamycin, 34 µg/mL chloramphenicol, 200 µM 5-aminolevulinic acid and 12.5 µM ferric ammonium citrate at 37 °C until an optical density (600 nm) of 0.9 was reached. Then, protein production was induced by the addition of 1 mM IPTG (final concentration) and the cultures were further grown overnight for 15-18 h at 20 °C. The cells were collected by centrifugation. The cell pellet was suspended 1:3 (w/v) in buffer C (50 mM Tris-HCl, pH 8, 150 mM NaCl), and the cells were lysed by passage through a French Pressure cell at 1200psi. The soluble protein fraction was obtained by centrifugation at 125,000 *g* for 1 h at 10 °C. NirC was purified by Strep tag affinity chromatography using a gravity flow column filled with Strep-Tactin XT 4Flow high capacity resin (IBA Lifesciences) and equilibrated with 5 CV buffer C. After loading the soluble protein fraction on the column, unbound proteins were washed off with 10 CV of buffer C. NirC was eluted from the column with 5-10 CV buffer BXT (IBA Biosciences). Finally, the NirC containing fractions were combined and the buffer was exchanged against NirC buffer (50 mM Tris-HCl, pH 8, 150 mM NaCl) by using a PD-10 desalting column (Cytiva). Protein concentrations were determined by the Bradford method with bovine serum albumin as the standard (Bio-Rad Laboratories).

Ethyl acetate extraction of tetrapyrroles

For the extraction of tetrapyrroles, the enzymatic reaction mixtures were stopped by the addition of 2% (v/v) concentrated HCl. Then, for each 250 µL of stopped assay mixture, 600 µL ethyl acetate were added, and the samples were vigorously mixed for 6 min using a Vortex-Genie mixer. Phase separation was achieved by centrifugation at 21,000 *g* for 10 min at 4 °C. The organic phase was transferred into a new Eppendorf tube and the ethyl acetate was

completely evaporated in a Concentrator Plus vacuum centrifuge. The dried pellets were stored at -20 °C until further use.

HPLC-UV/Vis analysis

HPLC-UV/Vis analysis was performed at 25 °C using a JASCO HPLC 2000 series system (Jasco, Gross-Umstadt, Germany) equipped with an MD-2015 diode array detector as described previously (Meyer et al. (2025), FEBS J., 292,5151). Briefly, the extracted and dried tetrapyrroles were dissolved in 100 µL of the initial condition of the HPLC analysis (10 µL methanol, 10 µL acetonitrile, 80 µL 1 M ammonium acetate, pH 5.2). The column (Equisil BDS C18, 250 x 4.6 mm, 5 µm (Dr. Maisch HPLC GmbH, Ammerbuch-Entringen, Germany)) was equilibrated with 80% 1 M ammonium acetate, pH 5.2 (solvent A), 10% methanol (solvent B) and 10% acetonitrile (solvent C) at a flow rate of 0.5 mL/min. After injection of 30 µL of sample, a linear gradient was applied reaching 60% A, 30% B, 10% C after 25 min and 90% B, 10% C within the next 15 min. These conditions were held for 20 min before returning to the initial conditions. The elution of tetrapyrroles was followed by recording the absorption at 385 nm (Meyer et al. (2025), FEBS J., 292, 5151).

HPLC-MS analysis

HPLC-MS analysis was carried out on a Thermo Fisher Scientific HPLC UltiMate 3000 System TSQ Quantum Access Max mass spectrometer adjusted to the scan range of m/z 200–1500 in positive ion mode; the target mass was set to m/z 800. Curtain gas pressure of 30 psi, ion spray voltage of 2500 V, and a temperature of 450 °C were employed during the analysis. Sheath gas pressure was 30 psi, and aux gas pressure was 5 psi. The collision energy was 10 V (Meyer et al. (2025), FEBS J., 292, 5151).

Structure prediction with Chai-1

The computational model of the NirF/DDDH-dilactone complex was generated by using the Chai-1 webtool (https://lab.chaidiscovery.com).

The input sequence of NirF (matching the sequence covered in the crystal structure) was:

PPLRGSGDLGVLIERADGSVQILDGTAKTSLARVEGLGDLSHASLVFSRDQRYAYVFGRDGGLTKLDLLAQRIDKRLI
QGGNSIGGAISQDGRLVAVSNYEPGGVKVFDSRTLELVAEIPATRLPGQDRNSRVVGLVDAPGQRFVFSLFDSGEI
WIADFSQGDTPHLTRFRDIGKQPYDALISPDGRYYMAGLFGEDGMAQLDLWHPERGVRRVLGDYGRGQRKLPVY
KMPHLEGWTIASDQAFVPAVGHHQVLVLDARDWKQTDAIDVAGQPVFVMTRPDDRQIWVNFAYPDNDKVQV
IDSETHEVIETLRPGPGVLHMEFSGRGDQVWISVRDADQLQVWDPYRLKRIGSLPARSPSGIFFSHRAQHIGL

The input file for the ligand was:

CC(C1=N/2)=C(CCC(O)=O)C2=C\C3=C(CCC(O)=O)C(C)=C(N3[Fe]4)/C=C5N=C(/C=C([C@@](C)(C6)[C@H
]/7OC6=O)\N4C7=C/1)[C@H]8[C@@]\5(CC(O8)=O)C

In the Chai-1 model, the regiochemistry at rings A and B of the DDDH-dilactone was inverted and was therefore corrected manually by using the Builder function in PyMOL. This did not result in clashes between protein and ligand.

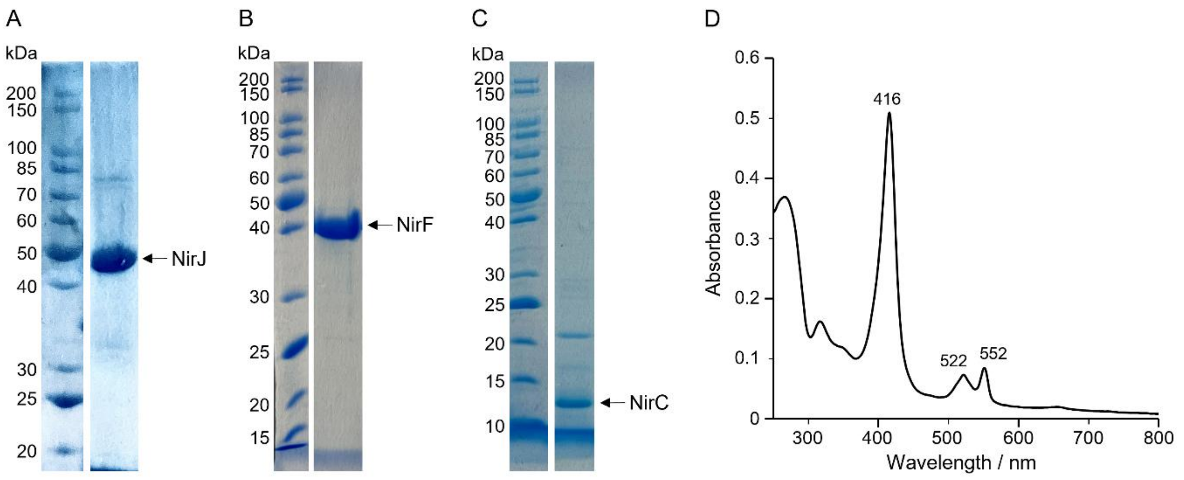

Figure S1. SDS-PAGE analysis of purified NirJ (A), NirF (B) and NirC (C), and UV/Vis absorption spectrum of purified NirC (D). The molecular weight standard is the Unstained Protein Standard, Broad Range (10-200 kDa) from New England Biolabs.
